# Relationship Between the Amount of Change in Echo Intensity and Young’s Modulus of the Soleus Muscle After Ankle Fracture Surgery

**DOI:** 10.7759/cureus.72624

**Published:** 2024-10-29

**Authors:** Hayato Miyasaka, Bungo Ebihara, Takashi Fukaya, Shigeki Kubota, Hirotaka Mutsuzaki

**Affiliations:** 1 Department of Rehabilitation, Tsuchiura Kyodo General Hospital, Tsuchiura, JPN; 2 Graduate School of Health Sciences, Ibaraki Prefectural University of Health Sciences, Ami, JPN; 3 Department of Physical Therapy, Faculty of Health Sciences, Tsukuba International University, Tsuchiura, JPN; 4 Department of Occupational Therapy, Faculty of Health and Medical Sciences, Ibaraki Prefectural University of Health Sciences Hospital, Ami, JPN; 5 Center for Medical Science, Ibaraki Prefectural University of Health Sciences, Ami, JPN; 6 Department of Orthopedic Surgery, Faculty of Health and Medical Sciences, Ibaraki Prefectural University of Health Sciences Hospital, Ami, JPN

**Keywords:** echo intensity, elastography, muscle quality, stiffness, young’s modulus

## Abstract

Objective: We aimed to clarify the relationship between the changes in echo intensity (EI) and Young’s modulus of the soleus (SOL) muscle after ankle fracture surgery.

Methods: Sixteen participants after ankle fracture surgery participated in this study (mean age: 46.8 ± 21.4 years). At three and five months after surgery, ankle range of motion (ROM), ankle strength, SOL muscle EI, and Young’s modulus were measured, and changes in values were calculated. The EI was measured using the B-mode and ImageJ software (National Institutes of Health, Bethesda, MD), and Young’s modulus was measured using shear wave elastography (SWE). The EI values corrected for subcutaneous fat thickness were calculated. Correlation and simple regression analysis were used to clarify the relationship between the amount of change in EI, the amount of change in Young’s modulus, and the amount of change in ankle ROM.

Results: Simple regression analysis showed that the amount of change in EI influenced the amount of change in Young’s modulus of the SOL muscle (r = 0.623; p = 0.010) and the amount of change in ankle dorsiflexion ROM with the knee flexed (r = -0.702; p = 0.002).

Conclusion: The change in EI of the SOL muscle affected the change in Young’s modulus after ankle fracture surgery. Clinically, changes in the EI may reflect changes in muscle stiffness.

## Introduction

Ankle fractures reduce the range of motion (ROM) of the ankle due to pain and muscle stiffness [1], which affects daily activities and return to work [1]. Immediate management of ankle fractures involves immobilization and weight restriction [2], and the soleus (SOL) muscle, which plantarflexes the ankle joint, may become stiff.

Shear wave elastography (SWE) quantifies the stiffness of individual muscles using Young’s modulus [3]. A change therein indicates a change in stiffness [4], and SWE can be used before and after an intervention to determine the effectiveness thereof and changes over time [5]. Therefore, SWE can be used to measure Young’s modulus over time to monitor changes in muscle stiffness. However, SWE is expensive, and a limited number of facilities can implement it [6].

Echo intensity (EI) using ultrasound imaging is used to estimate muscle quality; a higher EI indicates greater accumulation of non-contractile tissue, such as connective tissue and fat within the muscle [7-9]. After ankle fractures, non-contractile tissues may increase owing to immobilization and weight restriction. EI is related to various functions, including muscle stiffness [6,10] and muscle strength [11]. Therefore, muscle stiffness and strength are expected to improve with decreased EI. However, no relationship exists between changes in EI and muscle strength before and after strength training [12-14]. There are scattered reports of EI decline after strength training [15,16], and a consensus has not been reached. Therefore, changes in EI may reflect not only changes in muscle strength but also changes in muscle stiffness. Based on this hypothesis, muscle stiffness and ROM may improve as EI decreases. However, the relationship between the amount of EI change in the SOL muscle and Young’s modulus change after ankle fracture surgery is unclear. Therefore, we aimed to determine the relationship between the change in EI and the change in Young’s modulus of the SOL muscle after ankle fracture surgery. We hypothesized that as EI decreases, Young’s modulus decreases, ankle dorsiflexion ROM improves, and changes in EI may show changes in muscle stiffness over time and can be used to determine the effectiveness of the intervention.

## Materials and methods

Participants

The study was conducted between July 2022 and August 2024. We included patients who underwent open reduction and internal fixation (ORIF) for ankle fractures and who underwent physical therapy. We excluded patients with maximum ankle dorsiflexion ROM <10°, multiple fractures, open fractures, postoperative complications such as infection or deep vein thrombosis, and a history of neurological and orthopedic disease. All participants had their ankle joints fixed in a cast or splint for >1 week and were placed on crutches for >4 weeks postoperatively. Measurements were performed three and five months after ORIF.

Eighteen participants who met the criteria were examined three months postoperatively; two participants dropped out; therefore, 16 participants were included in the five-month postoperative measurements. Data were collected from medical records regarding age, gender, height, number of fractures [17,18], and Lauge-Hansen classification [19]; weights were measured on a digital scale, and body mass index (BMI) was calculated.

This study was approved by the institutional ethics committee of Tsuchiura Kyodo General Hospital, Tsuchiura, Japan (reference number: 2024FY32) and was conducted in compliance with the Declaration of Helsinki.

Echo intensity and Young’s modulus

All ultrasound measurements were performed using a 2-10 MHz linear transducer (Supersonic Imaging, Aix-en-Provence, France). The same physical therapist with eight years of experience in musculoskeletal ultrasound used the B-mode to evaluate EI and SWE to measure Young’s modulus. All ultrasound examinations were performed using long-axis muscle views, with room temperature maintained at 25°C [20]. The participants were instructed to relax during measurements to maintain their usual level of activity and abstain from impact activities two days before the measurements.

**Figure 1:**
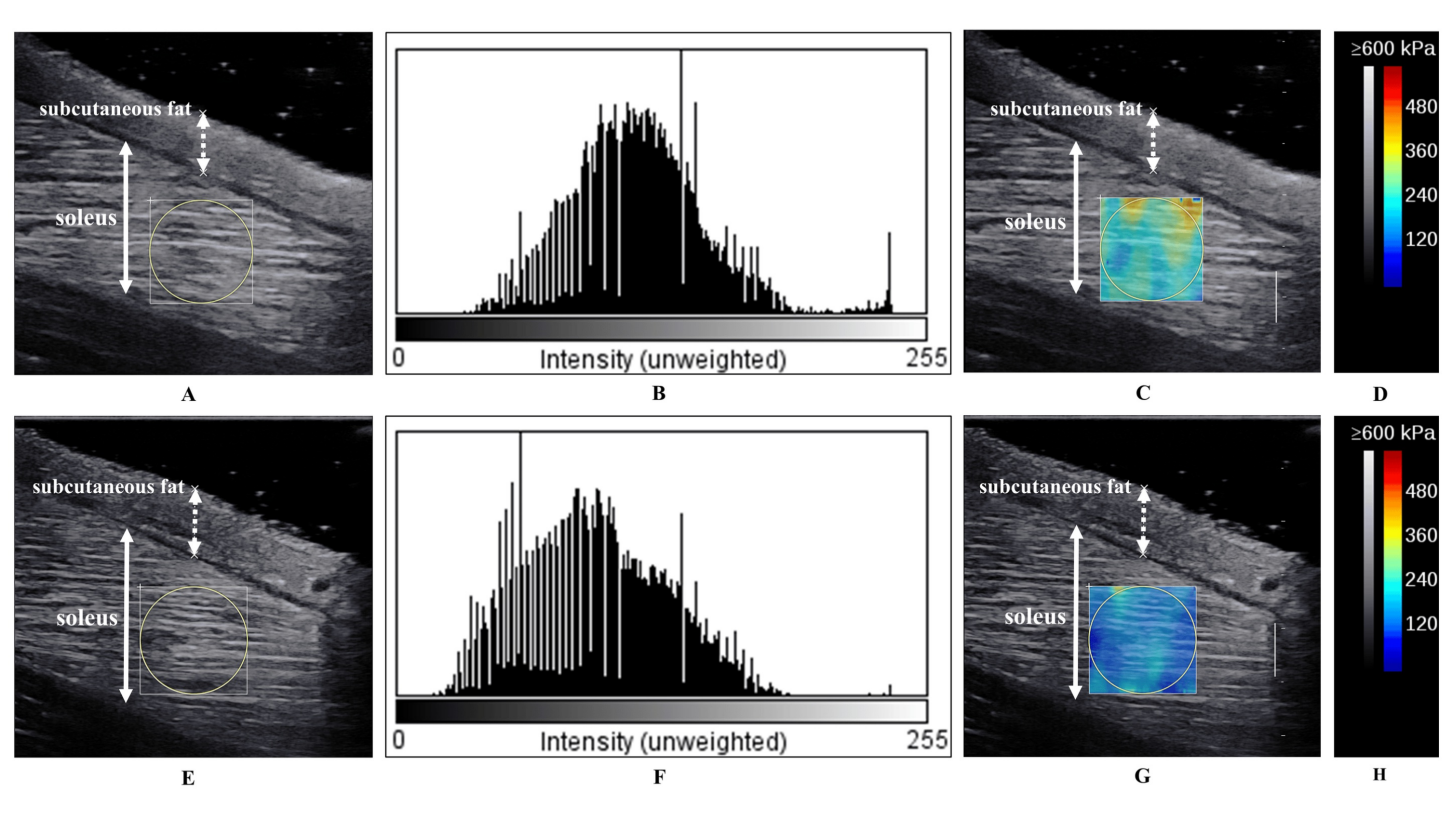
Typical example of the EI and Young’s modulus measurement (A) B-mode images of the SOL muscle three months after surgery; (B) values of 256 points calculated by Image J software three months after surgery; (C) SWE images of the SOL muscle three months after surgery; (D) the color scale; (E) B-mode images of the SOL muscle five months after surgery; (F) values of 256 points calculated by ImageJ software five months after surgery; (G) SWE images of the SOL muscle five months after surgery; (H) the color scale. The solid double arrows indicate the soleus muscle, and the dotted double arrows indicate the subcutaneous fat in (A), (C), (E), and (G). EI: echo intensity; SOL: soleus; SWE: shear wave elastography

All ultrasound measurements were performed according to procedures that demonstrated high intra-rater reliability [10]. Participants were measured with their knees bent at 90°, upper body supported on a table, ankles dorsiflexed at 10°, and kneeling [21]. The SOL muscle was measured in an area close to the gastrocnemius tendon transition [5], and B-mode horizontal axial images (focus depth, 2.0-4.0 cm in the middle of the SOL muscle; gain of 50%; and dynamic range of 72 dB) were used to confirm the muscle-tendon transition area, which was marked on the skin using a black pen. The EI was calculated as a 256-point value ranging from 0 (black) to 255 (white) by converting pixels in stored B-mode images to 8-bit grayscale values using ImageJ software (National Institutes of Health, Bethesda, MD) (Figure 1). The EI values corrected for subcutaneous fat thickness were calculated using the formula described by Müller et al. [22]:

Corrected EI [a.u.]=Uncorrected EI [a.u.]-5.0054×(subcutaneous fat thickness)^2^ [cm]+38.30836×subcutaneous fat thickness [cm].

Subcutaneous fat thickness was measured to a minimum of 0.1 cm using a digital measure in the ultrasound device (Figure 1). Corrected EI correlates better with Young’s modulus, ankle dorsiflexion ROM, and ankle plantar flexion strength than uncorrected EI [10]. Therefore, the corrected EI was used in this study.

To measure Young’s modulus, the parameter settings were as follows: mode of Opt penetration; range of approximately 0-600 kPa; region of interest of 10 mm in diameter was set near the center of the SOL muscle, and the same area was used for EI and Young’s modulus measurements (Figure 1).

Ankle ROM and strength

The ankle dorsiflexion and plantar flexion ROM were measured using a goniometer. First, the participants were placed in the supine position. Second, ankle dorsiflexion ROM was measured with the knee in the extended and bent positions and ankle plantar flexion ROM was measured with the knee in the bent position with the ankle dorsiflexed and plantarflexed as strongly as possible. Finally, the external phalanx, which was parallel to the plantar surface of the foot, served as the fulcrum for the measurement, and the fixation arm was positioned against the long axis of the fibula and measured at a minimum angle of 1°.

We measured ankle plantar and dorsiflexion muscle strength using a Biodex 3 dynamometer (Biodex Medical Systems, Shirley, NY). The participants bent their knees to 30° in a seated position and used straps to stabilize their lower body, thigh, and ankle muscles. The participants then performed bilateral isokinetic (concentric/concentric) ankle plantar flexion and dorsiflexion motions with two sets of five dynamic repetitions at an angular velocity of 60 °/s, 30 s apart [21]. We verbally encouraged the participants to make a maximum effort [23], measured peak torque at a minimum of 1 Nm, and calculated the peak torque/weight ratio.

Statistical analysis

The sample size was calculated (effect size = 0.40, α error = 0.05, power = 0.80) using G*Power 3.1 (Heinrich Heine University, Düsseldorf, Germany) [24, 25]; 16 participants were included. Measurements were compared three and five months postoperatively. Additionally, changes in the measured values were calculated. The degree of change was calculated by subtracting the measurements at three months postoperatively from the measurements at five months postoperatively. The distribution of each measurement and the amount of change were evaluated using the Shapiro-Wilk test. Normally distributed values were calculated as means and standard deviations; otherwise, medians and interquartile ranges were calculated. Paired t-tests and Wilcoxon signed-rank tests were used to compare the measurements at three and five months postoperatively. Pearson’s product moment and Spearman’s rank correlation coefficients were also calculated. Finally, a simple regression analysis was performed with Young’s modulus, ankle dorsiflexion ROM with the knee in the bent position, and ankle plantar flexion muscle strength as dependent variables and corrected EI as the independent variable. Statistical significance was set at p<0.05. All statistical analyses were performed using IBM SPSS Statistics version 29.0.2 (IBM Corp., Armonk, NY).

## Results

Participant characteristics

The participant characteristics are summarized in Table 1. The participants' mean age, height, weight, and BMI were 46.8 ± 21.4 years, 1.61 ± 0.09 m, 65.9 ± 14.9 kg, and 25.3 ± 4.2 kg/m^2^, respectively, and there were eight, six, and two class I, II, and III, respectively. The duration of ankle immobilization and crutch use after ORIF were 14.0 (7.0-14.0) and 48.6 ± 13.3 days, respectively. The time from ORIF to measurement was 91.9± 6.0 days at three months and 151.9± 8.2 days at five months postoperatively, respectively.

**Table 1:** Participants’ physical characteristics SER: supination-external rotation; PER: pronation external rotation; SA: supination-adduction Values are presented as mean ± standard deviation or n/n.

Parameters	n = 16
Age (years)	46.8 ± 21.4
Sex	
Male	9
Female	7
Height (m)	1.61 ± 0.09
Weight (kg)	65.9 ± 14.9
Body mass index (kg/m^2^)	25.3 ± 4.2
Number of fractures	
I	8
II	6
III	2
Lauge–Hansen classification	
SER	8
PER	3
SA	5

Comparisons after surgery

**Figure 2:**
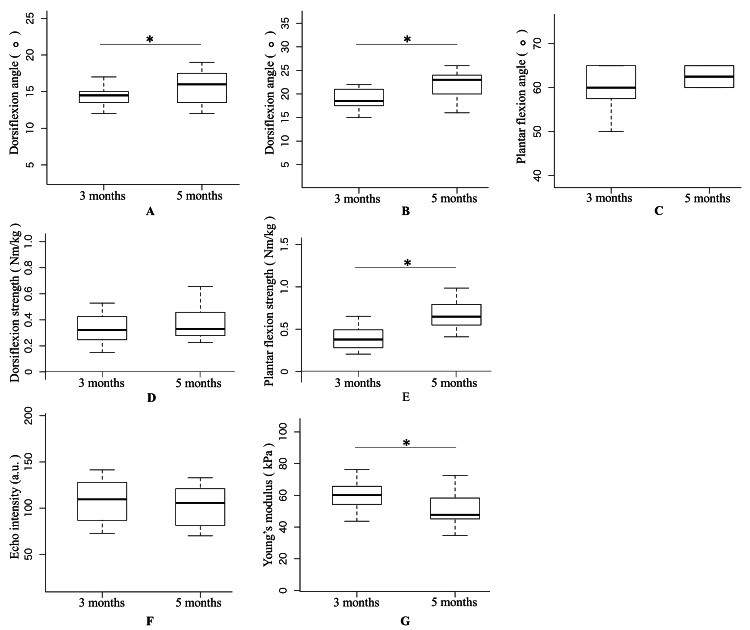
Comparison between three months and five months after surgery (A) ankle dorsiflexion ROM with the knee extended; (B) ankle dorsiflexion ROM with the knee flexed; (C) ankle plantar flexion ROM; (D) ankle dorsiflexion strength; (E) ankle plantar flexion strength; (F) echo intensity; (G) Young's modulus. * Significant differences were detected using paired t-tests five months after surgery compared with three months after surgery (p < 0.05). Thick lines within boxes and box borders indicate the median, 25^th^ percentile, and 75^th^ percentile, respectively.

The comparisons at three and five months postoperatively are summarized in Figure 2. At three and five months postoperatively, ankle dorsiflexion ROM with the knee extended was 14.1 ± 2.1° and 15.6 ± 2.3° (p=0.012, r=0.60), ankle dorsiflexion ROM with the knee flexed was 18.6 ± 2.8° and 22.1 ± 2.8° (p<0.001, r=0.84), ankle plantar flexion ROM was 59.7 ± 5.0° and 61.9 ± 4.0° (p=0.063, r=0.51), ankle dorsiflexion strength was 0.33 ± 0.11 Nm/kg and 0.37 ± 0.12 Nm/kg (p=0.081, r=0.44), ankle plantar flexion strength was 0.39 ± 0.13 Nm/kg and 0.68 ± 0.17 Nm/kg (p<0.001, r=0.91), EI was 107.6 ± 21.9 a.u. and 103.5 ± 20.7 a.u. (p=0.126, r=0.39), and Young's modulus was 59.8 ± 8.8 kPa and 50.6 ± 10.2 kPa (p<0.001, r=0.75), respectively.

Change in measurement values after surgery

The changes in measurements at three and five months postoperatively are summarized in Table 2. The changes in EI and Young's modulus of the SOL muscle, ankle dorsiflexion ROM with the knee extended, ankle dorsiflexion ROM with the knee flexed, and ankle plantar flexion strength were -4.1 ± 10.1 a.u. and -9.2 ± 8.5 kPa, 1.4 ± 2.0°, 3.4 ± 2.3°, and 0.29 ± 0.14 Nm/kg, respectively.

**Table 2:** Amount of change in measurement values three and five months after surgery Values are presented as mean ± standard deviation.

Measured values	Amount of change (n = 16)
Ankle range of motion (°)	
Dorsiflexion in the extended knee	1.4 ± 2.0
Dorsiflexion in the flexed knee	3.4 ± 2.3
Plantar flexion	2.2 ± 4.1
Ankle strength (Nm/kg)	
Dorsiflexion	0.04 ± 0.08
Plantar flexion	0.29 ± 0.14
Echo intensity (a.u.)	-4.1 ± 10.1
Young’s modulus (kPa)	-9.2 ± 8.5

Correlation coefficients

The correlations between the amount of change in EI and the amount of change in Young’s modulus of the SOL muscle and other measurement values are shown in Table 3. The amount of change in EI correlated with the amount of change in Young’s modulus of the SOL muscle (r = 0.623; p = 0.010), the amount of change in ankle dorsiflexion ROM in the flexed knee (r = -0.702; p = 0.002), and the amount of change in ankle plantar flexion strength (r = -0.747; p<0.001). The change in Young’s modulus of the SOL muscle correlated with the change in ankle dorsiflexion ROM in the flexed knee (r = -0.880; p < 0.001). The other measurement parameters were not significantly correlated (p > 0.05).

**Table 3:** Pearson’s correlation coefficients *Correlation was significant at p < 0.05; **Correlation was significant at p < 0.01

Measurements	Echo intensity		Young’s modulus	
	r	p-Value	r	p-Value
Ankle range of motion				
Dorsiflexion in the extended knee	-0.187	0.489	-0.129	0.633
Dorsiflexion in the flexed knee	-0.702	0.002**	-0.880	<0.001**
Plantar flexion	-0.238	0.376	-0.209	0.438
Ankle strength				
Dorsiflexion	-0.164	0.543	0.111	0.684
Plantar flexion	-0.747	<0.001**	-0.504	0.050
Young’s modulus	0.623	0.010*	-	-

Simple regression analysis

Table 4 presents the results of the simple regression analysis. The magnitude of change in the EI influenced the magnitude of change in Young’s modulus of the SOL muscle (standardized partial regression coefficient (β) = 0.623; p = 0.010), the ankle dorsiflexion ROM with the knee flexed (β = -0.702; p = 0.002), and the ankle plantar flexion strength (β = -0.747; p<0.001).

**Table 4:** Simple regression analysis B: regression coefficient; CI: confidence interval; ROM: range of motion; β: standardized regression coefficient

Dependent variables	Independent variables	Simple linear regression			
		B	95% CI of B	p-value	β
Young’s modulus	Echo intensity	0.522	0.147–0.898	0.010	0.623
Ankle dorsiflexion ROM in the flexed knee	Echo intensity	-0.158	-0.152 to -0.026	0.002	-0.702
Ankle plantar flexion strength	Echo intensity	-0.001	-0.015 to -0.005	<0.001	-0.747

## Discussion

The amount of change in EI influenced the change in Young’s modulus of the SOL muscle after ankle malleolus fracture surgery. The results of this study supported our hypotheses. Furthermore, the amount of change in EI also influenced the amount of change in ankle dorsiflexion ROM in the knee flexion position and the amount of change in ankle plantarflexion strength.

The EI is an indicator of muscle quality and reflects connective and fibrous tissues [9,26,27]. Most previous studies on muscle quality have focused on muscle strength. This is based on the idea that the higher the EI, the greater the accumulation of non-contractile tissues, such as connective tissue and fat, in the muscle [7-9]. Therefore, a relationship between improvements in EI and muscle strength is expected; however, no consensus has been reached on this to date. Cadore et al. [15] reported improvement in both muscle strength and EI in healthy young individuals who underwent isokinetic training for six weeks. Bassan et al. [12] reported an increase in muscle strength but no change in EI in healthy individuals who underwent resistance training for three weeks. Therefore, changes in EI may reflect changes in other factors in addition to muscle strength. An increase in connective tissue is associated with increased muscle stiffness [28]. Therefore, EI may affect muscle stiffness and ROM. The EI of the SOL muscle is positively correlated with Young’s modulus and may indicate muscle stiffness [10]. The EI of the SOL muscle is also negatively correlated with ankle dorsiflexion ROM in knee flexion and ankle plantar flexion strength [10]. However, it is unclear whether changes in EI over time are related to changes in muscle stiffness, ROM, and muscle strength. Here, we showed that a decrease in EI over time was associated with a decrease in Young’s modulus and an improvement in ankle dorsiflexion ROM. Increased muscle flexibility due to stretching may be associated with increased flexibility of connective tissues such as the fascia [29,30]. Therefore, the improvement in the flexibility of the connective tissue due to postoperative rehabilitation may contribute to the decrease in EI and Young’s modulus and the improvement in ROM.

Decreased EI in the SOL muscle was associated with improved ankle plantarflexion strength. The EI decreases after six weeks of strength training in healthy young individuals [15] and after eight weeks of strength training in patients with hip osteoarthritis [16]. Our results support those of previous studies. The EI also reflects intramuscular fat [11]. Therefore, the amount of change in EI may also affect the amount of change in muscle strength.

Clinically, changes in the EI can capture changes in muscle stiffness over time and may be useful to assess the effectiveness of interventions. This may be particularly useful to monitor the course of interventions in patients with immobilization and load restriction after ankle fracture surgery.

This study has some limitations. The participants were not controlled for fluid intake prior to EI measurement, and the SOL muscle activity was not monitored during these measurements. Finally, the partial quality of the SOL muscle was measured. Further research is required in this area.

## Conclusions

Simple regression analysis showed that the change in EI of the SOL muscle was related to the change in Young's modulus and the change in ankle dorsiflexion ROM with the knee flexed after ankle fracture surgery. Additionally, the change in EI also influenced the amount of change in ankle plantarflexion strength. Therefore, we suggest that the decrease in EI contributes to the improvement of muscle stiffness, ROM, and muscle strength. Clinically, changes in EI can capture changes in muscle stiffness over time and may help estimate intervention effects.
